# PROTOCOL: Domestic abuse interventions for mothers in or exiting prison: An evidence and gap map

**DOI:** 10.1002/cl2.1313

**Published:** 2023-03-02

**Authors:** Michaela Rogers, Kelly Lockwood, Elizabeth Speake, Fiona Campbell

**Affiliations:** ^1^ Department of Sociological Studies University of Sheffield Sheffield UK; ^2^ School of Health & Society University of Salford Salford UK; ^3^ CRESA, Sheffield Hallam University Sheffield UK; ^4^ School of Health and Related Research The University of Newcastle Sheffield UK

## Abstract

This is the protocol for a evidence and gap map. The objectives are as follows: to identify existing research and gaps in evidence according to the types of interventions, settings, study design and outcomes; to use the EGM findings to inform subsequent systematic reviews and to identify gaps in evidence to inform future research, policy or practice.

## 1 BACKGROUND

### 1.1 Introduction

#### 1.1.1 The problem, condition or issue

Around 714,000 women and girls are held in penal institutions throughout the world, accounting for around 7% of the global prison population (World Prison Brief, 2017). Although they remain a minority in the global prison population, the number of women and girls in prison has increased, in all continents, by about 50% since the year 2000 (World Prison Brief, 2017). A UK‐based review of women's imprisonment, the Corston Report (Home Office, 2007) contributed to a greater recognition worldwide that there needs to be a distinct approach to women in prison (Finer, 2020). Chartrand and Kilty (Chartrand & Kilty, 2017) note that the most significant influence of the report in Canada relates to recognition of the need for integrated holistic and gender sensitive responses to women who encounter the criminal justice system. Similarly, in Australia, Stubbs and Baldry (Stubbs & Baldry, 2017) note how the report revived interest and consequent pressure from advocates, campaigners, and prison staff to transform women's prisons with particular attention to the structural issues leading to the imprisonment of women. The Corston Report led to the creation of the United Nations Rules for the Treatment of Female Prisoners and Non‐Custodial Measures for Women Offenders (known as the ‘Bangkok Rules’) adopted by the UN General Assembly in 2010 (UNODC, 2014). The Bangkok Rules is a set of 70 principles for the gender‐sensitive support of female offenders and prisoners. The rules also advocated for the provision of more community‐based alternatives to custody. However, 10 years after their adoption, an analysis of the global female prison population found a global increase, not a decrease, of 105,000 more women in prison (Prison Reform International, 2020).

Estimates of women in prison who are mothers are mostly high (Prison Reform Trust, 2017) although international comparisons are difficult owing to the differences in and limited reporting mechanisms and variations in definitions of ‘mother’ and who constitutes a ‘child’ (Codd, 2020). For example, in Finland, Enroos (2011) identifies a lack of systematic information with the parenting status unrecorded for 73% of prisoners. Equally, Codd (2020) notes that mothers may be unwilling to disclose that they have children owing to concerns as to the outcomes, including child protection intervention. Therefore, much of the available data is based on estimates. In America, it is estimated that between 65% and 80% of women in prison are mothers of minor children (Casey‐Acevedo et al., 2004). In Australia, it is estimated that almost half of women in prison have at least one child aged under 16 years (Newman et al., 2011). However, significant variations in terms of ethnicity are identified with Sullivan et al. (Sullivan et al., 2019) identifying that more than 80% of Aboriginal women in prison in Australia are mothers. Codd (2020) also notes that the numbers of children experiencing the imprisonment of a mother varies, reflecting the average birth rate per mother in each country.

Separation from their children may be especially difficult for women in prison who have been victims of violence (Casey‐Acevedo et al., 2004). This is significant as women in prison tend to have complex needs and multiple disadvantages. For example, in England and Wales, the Ministry of Justice (Ministry of Justice, 2012) identify women in prison as being more likely to have experienced: trauma throughout their lifetime; physical, emotional and/or sexual abuse as children; state or public care arrangements as a child; and to have witnessed violence in the home. In Spain, Valenzuela and Alcázar‐Campos (2019) draw on research to estimate that around 50% of women in prison have experienced domestic violence and abuse (DVA). In America, research indicates that at 70%–90%, women in prison are significantly more likely to have experienced DVA in their pre‐prison relationships in comparison to women in the general population at around 36% (Jones, 2020). The Prison Reform Trust (2017) indicates that around 57% of women in prison in England and Wales have experienced DVA. Day and Gill, 2020 note that DVA manifests in various ways in socioeconomic groups and cultural contexts, and experiences of abuse and victimhood are shaped by victims' intersectional identities and locations. They go on to suggest that there are additional challenges for BME women that compound their experiences of DVA. Recognising and responding to these structural inequalities is particularly relevant when exploring DVA in relation to women in prison, as this subgroup are some of the most marginalised women in society; women of colour and those from lower socioeconomic backgrounds are over‐represented within the women's prison population in most western societies.

Recent research in the United States (US) has explored the pathways between women's experiences of DVA and their criminalisation. Jones (Jones, 2020) identified how women's imprisonment was often owing to them reportedly being forced by an abusive partner to either participate in an offence or take responsibility for an offence they had not committed. Jones (Jones, 2020) found that women may also engage in violent behaviour as a means of self‐defence; whilst Durfee and Goodmark (Durfee and Goodmark, 2021) found that male abusers may also file a complaint, which they termed ‘cross‐filings’, to lever the legal system, leaving women more vulnerable to arrest, charge and potential imprisonment. For women in prison, DVA can inform how they engage with and respond to interventions (Ministry of Justice, 2018). In a European‐wide study exploring women prisoners, mental health, violence and abuse, Macdonald (2013) identified that those who had experienced DVA found prison life more difficult to manage. Macdonald (Macdonald, 2013) indicated that for many women the prison environment was infantilising, removing autonomy and demanding complicity, which was reminiscent of their previous experiences of abuse.

This was echoed in a UK study by Crewe et al. (2017), which found that loss of power, autonomy and control was a significant challenge for women in prison. Women struggled with loss of control of their intimate daily practices, nutrition, clothing and ability to maintain contact with their children. They suggested that the feelings of powerlessness reproduced many of the dynamics of abuse they had previously experienced. DVA may also affect a mother's experience of and responses to imprisonment. Contact may be restricted or denied by the carer of children of a mother in prison, as a means of further punishing or controlling the mother (Flynn, 2014). Equally, contact may be restricted or limited if social services intervention has occurred before the mother's sentence. The combined experiences of DVA, imprisonment, and separation from their children can be traumatic, trapping mothers in a ‘vicious cycle of victimisation and criminal activity’ (Prison Reform Trust, 2017, p. 4). Mothers in prison can experience shame, hopelessness and failure which can trigger a return to self‐destructive behaviours which, after release from prison, may impact successful resettlement and reunification (Baldwin, 2017; Macdonald, 2013). In the United States, Hart‐Shuford et al. (Shuford et al., 2018) found the experience of physical partner abuse to be a significant risk factor for depression during release from prison, having implications for successful resettlement. Similarly, commenting on the Spanish context, Valenzuela and Alcazar‐Campos (Valenzuela‐Vela and Alcazar‐Campos, 2019) argue that with limited support on release, many women will have to return to their violent partners. It is important to bear in mind that the limited evidence in this area illustrates a correlation between DVA and maternal incarceration rather than DVA being a cause leading to criminal justice involvement.

#### 1.1.2 The interventions

This evidence and gap map (EGM) is focused on interventions in which the primary aim is to address historic or current domestic violence and abuse and its impacts *and* which are targeted at or available to mothers who are in, exiting, or have recently exited prison (within a 12‐month timeframe and who are subsequently released on license and subject to mandatory license conditions, such as attending appointments with an offender manager). Globally, the body of evidence on empirically validated interventions for DVA is sparse in terms of women in the criminal justice system and particularly for different groups of victim‐survivors of DVA (Trabold et al., 2020). Interventions included in this EGM will therefore comprise a variety of services and programmes which can occur in any setting including, but not limited to, criminal justice settings, domestic and sexual violence support services, women's centres, accommodation‐based services, outreach services, and remotely via technology platforms. The content, mode and manner of delivery and length of the intervention may differ in each of the studies to be included as there is no standard evidence‐based programme for addressing DVA for women or mothers in, exiting, or recently released from prison. The content of an intervention, for example, might be based around challenging beliefs about DVA, building self‐esteem and resilience, or developing positive relationship skills. Interventions will therefore also likely be diverse in terms of theoretical underpinnings. It is important to identify relevant theory/theories which underpin interventions and it is important to understand how the intervention works in another context, within a particular setting or with a particular subgroup of mothers (women of colour for example). Models of care may also differ depending on the service provider (including statutory bodies and charities). An indicative list of the types of interventions to be included in the review is shown in Table 1.

**Table 1:** Typology of interventions.

Type of intervention	Description	Example
Advocacy	Interventions inform victim‐survivors of their rights, and the services available to them. Activities might include emotional and practical support, impartial advice, general information relating to criminal justice pathways and safety planning.	Domestic Violence Advocates or Advisors
Psychosocial and skill‐building	Group or individual programmes that use education and training to improve awareness, knowledge, and/or skills related to DVA or parenting among a group of individuals (e.g., female offenders, mothers, etc).	The Freedom Programme You and Me, Mum
Therapeutic support	Group or individual counselling that provides victim‐survivors of violence with emotional, psychological and social support.	Trauma‐informed counselling, Cognitive Behavioural Therapy
Outreach	Outreach support for victim‐survivors deemed to be at low‐medium risk of harm from DVA. Interventions include home visits, emotional and practical support, impartial advice, general information relating to criminal justice pathways and safety planning. Interventions inform victim‐survivors of their rights, and the services available to them, and improve knowledge of the different forms, risk factors, and consequences of violence.	Outreach visits, home visitation
Peer support	Group or individual support where people use their own experiences to help another. Support includes sharing knowledge or experiences and includes emotional, social or practical help. It commonly refers to an initiative consisting of trained supporters, and can take a number of forms such as peer mentoring, reflective listening, or counselling.	Local mentoring schemes, peer groups
Technology‐based intervention	Support provided using mobile, wireless and web‐based platforms, such as through smartphone apps, text messaging, and online support.	isafe, iCan Plan for Safety

##### How the intervention might work

A DVA intervention may work in the following ways:

- Offering ongoing social and emotional support.
- Increasing individualised safety planning and risk assessment of frequency, severity, and types of violence and abuse.
- Enabling skills in conflict resolution.
- Providing impartial information and education to improve informed decision‐making.
- Improving mental health outcomes and general wellbeing.

The mechanism for change may vary, such as increased social and emotional support leading to reduced DVA. Other interventions might demonstrate outcomes by recording victim‐survivor self‐reporting of mental health outcomes. The mechanism for improvement of the mental health of victim‐survivors derives from the practical and emotional support provided to alleviate the effects of DVA and support safety planning (e.g., in advocacy intervention) (Ferrari et al., 2018). In a complex intervention, such as trauma‐focussed counselling or therapy, which is grounded in dialogue (i.e., a talking therapy), the mechanism for change might be the opportunity for the victim‐survivor to recognise prior experiences as abuse and, as such, can be experienced as empowering (Petrillo, 2021). The proximal change may be increased self‐esteem and confidence leading to changes in decision‐making and subsequently reduced DVA.

The theoretical foundation for an intervention could draw from a number of approaches, including (but not limited to) feminist principles, empowerment theory, psychosocial theory, and strengths‐ or asset‐based approaches. The UK's National Offender Management Service (NOMS) identifies several types of intervention that are likely to be effective for improving the mental health of female offenders and managing the impacts of DVA, including: advocacy interventions; social support; mentoring; trauma‐focussed cognitive‐behavioural programmes; and short‐term trauma‐focussed counselling (NOMS, 2015, p. 16). A commonly found theoretical model underpinning DVA interventions is the Duluth Model. The Duluth Model (named after the US city where it was developed) is built using feminist theory which centres the notion that male violence against women and children results from gender inequality and male dominance. It embeds a conceptualisation of power and control at the heart of DVA perpetration. The Duluth Model, therefore, is predicated on putting victim‐survivor safety and perpetrator accountability at the centre of all procedures and interventions (Taylor & Sullivan, 2008). The model requires a coordinated, multi‐agency approach which develops connection and consistency between agencies to ensure a robust safety net for victim‐survivors (Taylor & Sullivan, 2008). Tools developed by the coordinating body of the Duluth Model, Domestic Abuse Intervention Programs (DAIP) have been widely replicated for use by intervention programmes across the United States and internationally. Such programmes include information on mothering and nurturing children after DVA. The work of Evan Stark (Stark, 2007) on coercive control and the gender inequality which underlies DVA also informs many interventions. Stark highlights the impact of non‐physical, as well as physical, abuse and the primacy of intimidation, isolation and control as a patterning of abusive behaviour arguing that intervention models based on an incident‐specific understanding of DVA obscures the major components, dynamics and effects of abuse (Stark, 2007, pp. 9–10).

#### 1.1.3 Why it is important to develop the EGM

Research shows that the female prison population is increasing (World Prison Brief, 2017). A significant proportion has current or historic trauma and experiences DVA and a similar proportion are mothers to dependent children (Codd, 2020; McCauley et al., 2020). Research also shows us that DVA and prison disrupt mothering and the mother‐child relationship (Lockwood, 2017). This EGM will describe DVA interventions in prison and for mothers on their release from prison looking at evidence over a wide range of outcomes. This is important as research suggests that understanding of the intersection of mothering, women's criminalisation and DVA is little understood (Roberts, 2019). It is also important as no review to‐date has specifically focused on DVA interventions for mothers who are in prison or recently exited.

An intersectional lens will explore mothers as a diverse group paying attention to differences in social characteristics and backgrounds (e.g., ethnicity, asylum and migration status, age and disability). The analysis and our final reporting will be mindful of impacts and changes to the policy and practice environment due to Covid‐19 (the global pandemic).

This EGM will generate more informed understanding of the existing support available to mothers in, exiting or recently released from prison, and what is missing (Prison Reform Trust, 2017). The social benefits of generating a better understanding of the intersection of DVA and contact with the criminal justice system are numerous including better targeted support, improved engagement with services, reduced offending, reduced rates of DVA and better outcomes for children impacted by maternal imprisonment.

## 2 OBJECTIVES

The primary objective of this EGM is to present the existing research on the impact of interventions that address domestic abuse for mothers who are in, exiting, or have recently been released from prison, mapping what *types* of interventions have been evaluated, what study designs have been used, and what outcomes have been measured. This will provide the foundation for subsequent systematic reviews that will explore what types of interventions work, for *whom*, and in which *contexts*. It will describe the quality of available evidence, highlight the gaps to inform future research priorities and to enable a more comprehensive understanding of the available knowledge of this topic. Specifically, this EGM will include the following objectives:

- To identify existing research and gaps in evidence according to the types of interventions, settings, study design and outcomes.
- To use the EGM findings to inform subsequent systematic reviews.
- To identify gaps in evidence to inform future research, policy or practice.

## 3 METHODS

### 3.1 EGM: Definition and purpose

Saran and White (2018) define an EGM as ‘a systematic [visual] presentation of the availability of relevant evidence for a particular policy domain. The evidence is identified by a search following a pre‐specified, published search protocol. The map may be accompanied by a descriptive report to summarize the evidence for stakeholders such as researchers, research commissioners, policy makers, and practitioners’ (p. 11). An important distinction to note is that EGMs summarise what evidence exists but not what the evidence says. For instance, an EGM describes studies in a particular policy area in terms of outcomes and interventions.

EGMs are useful in identifying evidence gaps, collections of studies for review, and an EGM will identify where there is a need for more research or rigorous evaluation. They can be used to generate higher‐level evidence products such as guidelines or in the development of interventions. Traditional methods adopted for EGMs include a focus on quantitative data only. However, there are EGMs that set a new precedent as these include different research designs and qualitative data (Tallent et al., 2022) as this EGM will do (see ‘Treatment of qualitative research’).

### 3.2 Framework development and scope

Development of the framework is considered to be the most important, and can be, the most difficult part of developing an evidence map (White et al., 2020). The framework provides the structure of the EGM and is one of the first activities and is a primary resource in the development of search strategy, screening and coding tools (Apunyo et al., 2022). The framework for this EGM will follow a typical matrix of intervention categories (rows) and outcomes (columns). Where reported, we will extract information on service user perspectives as well as process and implementation factors.

### 3.3 Stakeholder engagement

The framework was developed following consultation with an expert Advisory Group constituted by members from relevant fields in the UK. Specifically, members included:

- Research manager from a national domestic abuse charity.
- Member of a national campaigning prison charity.
- Research Support Officer from the Ministry of Justice.
- Regional manager from a women's alliance organisation.
- Research Manager for the Justice arm of a national funding body.

Members of the group commented on the draft protocol leading to refinement and clarification to the protocol as well as redefined outcomes of interest. The intervention and outcome framework was developed from a review of literature and examination of key policy documents.

### 3.4 Conceptual framework

**Figure 1:**
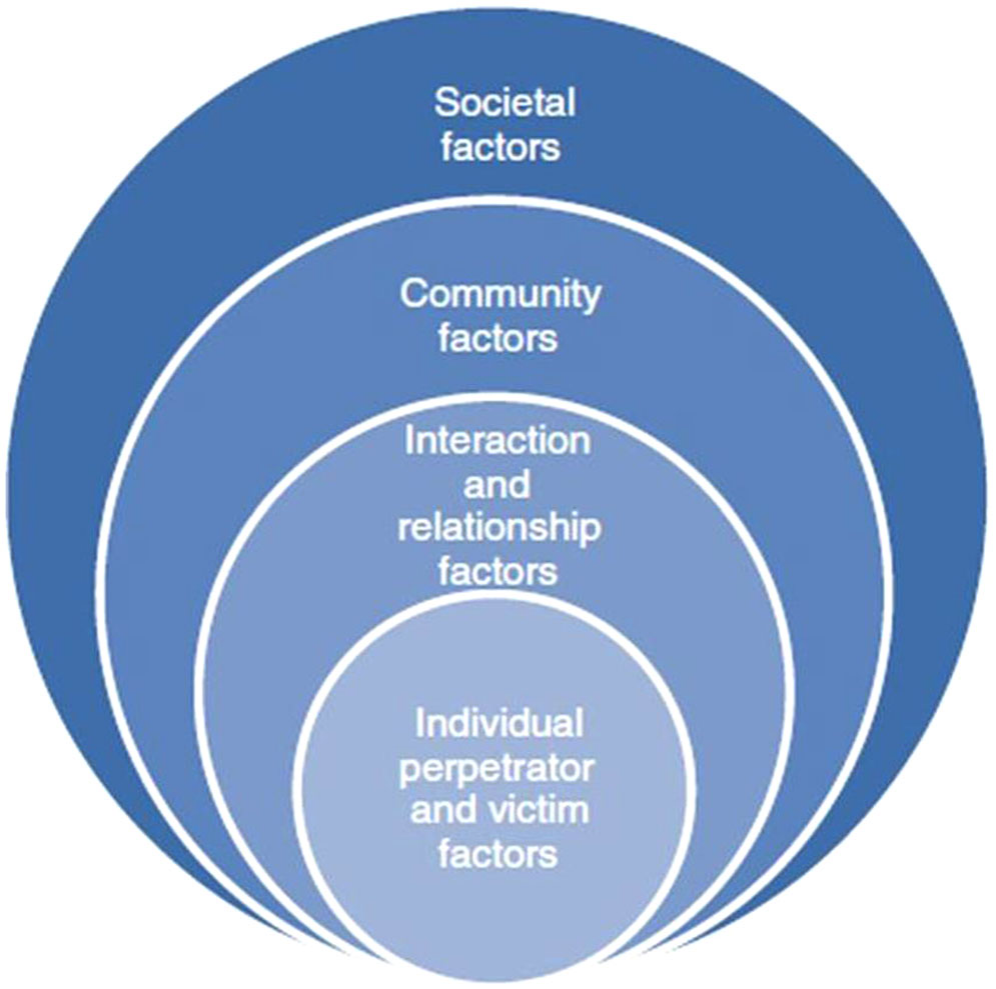
Sociological ecological model.

The conceptual underpinning of the development of the framework draws upon t*he socio‐ecological model* which helps to identify and explain the multidimensional aspects of domestic abuse (Bronfenbrenner, 1979), in particular to help map interventions and outcomes across individual, community and society (Figure 1). The socio‐ecological model is commonly used in interpersonal violence research.

The socio‐ecological approach proposes that human beings are embedded in nested systems related to context and progressively adapt to accommodate to their environment over time. Individuals are affected by, and in turn affect their environments. In addition, reciprocal causation is present, which means individual behaviour moulds, and is moulded by, environment (Phelan & Kirwan, 2020). Thus, the socio‐ecological model allows an integration of individual and environmental factors to enable an examination of interventions and outcomes within complex systems.

### 3.5 Dimensions

The outcomes axis of the framework for this EGM will be based on the following dimensions:

- DVA victimisation or revictimisation
- Agency and self‐efficacy in relation to relationships, conflict and abuse
- Social and gender norms in relation to relationships, conflict and abuse
- Risk and safety planning
- Reoffending rates
- Wellbeing (general or overall mental/emotional/psychological health)
- Substance misuse
- Safer accommodation
- Mothering and parenting practice
- Mother–child relationship
- Acceptability of interventions

We will code any information related to process and implementation factors noted in the studies. We will be involving our stakeholders as we refine our framework. The socio‐ecological model will inform our framework. Interventions will be categorised on level of intervention (women, criminal justice system, wider society). We will also use the theoretical mechanism underpinning their action to further group the types of interventions. Further categories in our framework will include the settings in which it was delivered, the agencies involved, and the target (DVA or DVA and another factor). We will be undertaking further stakeholder engagement in the process of refining the framework. We may include filters such as: age, socioeconomic status, nature of the abuse, age of children as guided. We will also include country as a coding domain.

#### 3.5.1 Types of study design

The EGM will include primary studies and evaluations of the effectiveness of interventions that aim to address DVA experienced by mothers in or exiting prison (see Table 2). An impact evaluation is defined as any intervention evaluation that uses qualitative, quantitative or mixed methods design applied to experimental or observational data that measures the effect of an intervention compared to what would happen to the same group in the absence of that intervention.

**Table 2:** PICO Framework.

P	Population	Women (aged 16 years or older) who identify as mothers, as victim‐survivors of DVA, and who are offenders either in, exiting, or recently released from prison.
I/E	intervention/exposure	Any intervention identifiable in our typology that addresses DVA (physical, sexual, psychological or emotional abuse, financial and material abuse), current or previous, or mothering.
C	Comparator	No intervention.
O	Outcome	DVA victimisation; social and relationship norms; increase in self‐efficacy in relation to relationships, conflict and abuse; risk and safety planning; parenting; mother‐child relationships; health including substance misuse and mental health.

To include both completed and ongoing impact evaluations and systematic reviews, we will search trial registries and protocols. Authors will be contacted for a timescale of their project to ascertain if the timescale of data collection is within or outside the timescale for this EGM. If outside of this timescale, it will not be included in the current EGM, but reference to the study will be noted for future updates of the EGM.

##### Inclusion criteria

1. Randomised controlled trial (RCTs): Studies where participants are randomly allocated to control and intervention conditions.
2. Quasi‐RCTs: Studies in which participants are allocated to control and intervention groups through a quasi‐random approach (e.g., through the order of recruitment).
3. Quasi‐experimental studies: Studies where participants in the intervention and control conditions are assigned to conditions in a non‐random manner (e.g., study participants self‐select or are already located in groups).
4. Mixed method studies of all design types (e.g., explanatory, or exploratory design) will be included. These are studies which include both a quantitative and qualitative component.
5. Qualitative studies with approaches to research design such as phenomenology, ethnography, narrative and grounded theory, will be eligible for inclusion.

Any limitations, biases, ethical or safety issues associated with any study design will be examined in relation to their potential impact on the effectiveness or acceptability of the intervention and validity of the study's findings. We will include pilot or proof‐of‐concept studies or impact/process evaluations including quantitative or qualitative data. We will not limit our inclusion criteria to RCTs or comparator studies. Where we will be including comparative studies, we will consider any type of comparator including; no intervention and an alternative intervention.

Studies will be full‐text accessible, deposited in published or unpublished repositories, with no date limitations. Where the full‐text cannot be located, these will not be excluded but listed in ‘studies awaiting classification’. If studies are published in language other than English, we will attempt to assess eligibility, access a full translation or include (if relevant) by using the Google Translate tool.

#### 3.5.2 Treatment of qualitative research

This EGM will include qualitative research if it meets the inclusion criteria and fits within the intervention‐outcomes framework; that is, we will include a document if it is a qualitative empirical study focused on an eligible intervention. This will include projects that are entirely qualitative or ones which are part of a mixed‐methods design. Qualitative data collection such as interviews, focus groups, observation, and participatory designs will be considered in the EGM. This includes qualitative research where data is collected on the perspectives and experiences of service users or service providers. This research may relate to the barriers or facilitators to the effectiveness of interventions and also the accessibility and acceptability of interventions.

We will consider the following publication types as ineligible and these will therefore be excluded: (1) opinion pieces; (2) commentaries; (3) editorials; (4) debates; (5) project implementation guidelines; (6) other reflective non‐research based reports and (7) systematic and non‐systematic reviews (albeit if systematic and non‐systematic reviews are sourced, these would be used for citation searching).

#### 3.5.3 Types of intervention/problem

This EGM will examine interventions aimed at distinct needs relating to abuse experiences, impacts and trauma. As such, intervention types will include individual, group or programme‐based interventions (as per the typology detailed in Table 1) addressing DVA and its impacts. Interventions may use various forms of delivery approaches, including different approaches to facilitation (professional or peer‐led) and intervention characteristics such as this will be coded within the framework.

#### 3.5.4 Types of population

The population of interest is limited to study participants who are mothers either in, exiting or recently released from prison (within a 12‐month timeframe and who are subsequently on probation), with current or past experience of DVA. The United Nations (n.d., online) defined DVA as ‘a pattern of behavior in any relationship that is used to gain or maintain power and control over an intimate partner. Abuse is physical, sexual, emotional, economic or psychological actions or threats of actions that influence another person. This includes any behaviors that frighten, intimidate, terrorize, manipulate, hurt, humiliate, blame, injure, or wound someone’. In recognition that the age of criminal liability and age that a female offender may be sent to prison will differ across countries, we will not use a minimum age restriction. We will include studies where participants reflect a range of ethnicities, sexual and gender minorities, and ages, as well as pregnant or single mothers, and mothers from low or middle‐income countries. We will include studies which include samples of females and mothers combined. We shall make it clear in our coding framework so that studies with mixed populations can be differentiated from other studies.

We will exclude: studies that evaluate interventions that do not address DVA with participants; perpetrator focused studies; studies that do not include female offenders; studies that focus on violence and assault by strangers, or where it is not possible to discern the relationship between victim‐survivor and perpetrator; studies that are based in settings outside of our inclusion criteria in terms of the participant group. Studies will be excluded if the extent of focus on mothers in, exiting, or recently released from prison is unclear. If a study meets the inclusion criteria, but only a subset of the population is eligible for inclusion (e.g., some participants are in prison and others on community‐based orders), we will include only the eligible population if the data are disaggregated. Where the data are combined within the study, we will contact study authors to request the relevant disaggregated data. If we are unable to obtain such data, we will exclude the study.

#### 3.5.5 Types of outcome measures

This EGM aims to scope the impact of DVA interventions for mothers in or exiting prison. Outcome measures of interest are:

- DVA victimisation or revictimisation (e.g., self‐reported experience of DVA, official data such as police calls‐for‐service)
- Agency and self‐efficacy in relation to relationships, conflict and abuse (recorded in intervention evaluation, or self‐assessment)
- Social and gender norms in relation to relationships, conflict and abuse (recorded in intervention evaluation, or self‐assessment)
- Risk and safety planning (prison records, prison or probation case management records)
- Reoffending rates (recorded as crime to the police, probation records, or self‐reported offending following exit from prison)
- Wellbeing (general or overall mental/emotional/psychological health)
- Substance misuse (changes in use recorded in prison records, or through self‐reports to intervention facilitators/self‐assessment)
- Safer accommodation (permanent housing, supported accommodation)
- Mothering and parenting practice (self reports of increased contact with children, improved parenting skills recorded in self‐assessment)
- Mother–child relationship (self reports of increased contact leading to change in relationship, self‐assessment, or recorded in intervention evaluation)

Outcomes may be measured through validated or standardised instruments (e.g., an Outcome Star, an outcome measure that uses scaling techniques (e.g., 1–10) undertaken by a professional or through self‐assessment) or other non‐standardised tools (e.g., self‐reports). In the case of qualitative research, we will include individual, narrative accounts that report perspectives and/or experiences in relation to any aspects of DVA in the target population, including, but not limited to, those being measured quantitatively. We will document any unintended adverse events reported, whether quantified or reported qualitatively, and code into the EGM.

##### Types of location/situation

No limitations will be placed on the country of study; for example, low and middle‐income countries.

##### Types of settings

Interventions may occur in prison or be community‐based after recipients have exited prison. Community‐based settings include criminal justice settings (e.g., prison, or the probation service for women subject to mandatory license conditions, such as attending appointments with an offender manager), domestic and sexual violence support services, women's centres, accommodation‐based services, outreach services and other community‐based settings.

### 3.6 Search methods and sources

This EGM will search for and include completed primary studies and evaluations. We will include published studies with no language restrictions to minimise publication bias. For studies written in a language other than English, we will attempt to obtain a complete translation or use Google Translate. We will seek advice from the subject librarians within the University library.

#### 3.6.1 Search terms

Keywords and terms have been derived from the scoping search and researcher expertise in the subject fields. Searches in each database will be expanded or restricted using Boolean operators (e.g., OR, AND), wildcards (e.g.,?), truncations (e. Including *) and by using limiting commands to narrow the results (e.g., ‘year of publication’). This will ensure search precision and sensitivity. An example of a search using the Boolean configuration for ‘intervention’ AND ‘domestic violence and abuse’ AND ‘women’ AND ‘imprisonment’ is provided in Table 3.

**Table 3:** Example search.

Intervention/Evaluation	DVA	Women/Maternal	Imprisonment
TS = (cohort* OR ‘cross‐sectional’ OR effect* OR efficac* OR evaluat* OR evidence OR experiment* OR impact* OR interven* OR ‘mixed‐method*’ OR ‘mixed method*’ OR prevent* OR program* OR qualitative OR ‘quasi experimen*’ OR ‘quasi‐experiment*’ OR ‘quasi‐random*’ OR ‘quasi random*’ OR ‘quasi RCT’ OR ‘quasi‐rct’ OR random* OR response* OR service* OR support* OR therap* OR treatment* OR trial* OR ‘what works’)	TS = (‘coercive control’ OR ‘intimate terrorism’ OR ‘violence against women’ OR ‘battered wom?n’) OR TS = (gender* NEAR/3 violen*) OR TS = (sex* NEAR/3 abus) TS = (sex* NEAR/3 assault) OR TS = (sex* NEAR/3 violen*) OR TS = (couple* NEAR/3 abus*) OR TS = (couple* NEAR/3 assault*) OR TS = (couple* NEAR/3 violen*) OR TS = (domestic* NEAR/3 abus*) OR TS = (domestic* NEAR/3 assault*) OR TS = (domestic* NEAR/3 violen*) OR TS = (intimate* NEAR/3 abus*) OR TS = (intimate* NEAR/3 assault*) OR TS = (intimate* NEAR/3 violen*) OR TS = (interpersonal* NEAR/3 abus*) OR TS = (interpersonal* NEAR/3 assault*) OR TS = (interpersonal* NEAR/3 violen*) OR TS = (partner* NEAR/3 abus*) OR TS = (partner* NEAR/3 assault*) OR TS = (partner* NEAR/3 violen*) OR TS = (relation* NEAR/3 abus*) OR TS = (relation* NEAR/3 assault*) OR TS = (relation* NEAR/3 violen*) OR TS = (spous* NEAR/3 abus*) OR TS = (spous* NEAR/3 assault*) OR TS = (spous* NEAR/3 violen*)	TS = (mother* OR maternal OR women OR female* OR wife OR wives)	TS = (correction* OR criminal* OR custod* OR detain* OR detention* OR felon* OR gaol* OR imprison* OR incarcerat* OR inmate* OR jail* OR offender* OR prison*)

#### 3.6.2 Search sources

Relevant studies will be identified through searches in electronic databases, governmental and grey literature repositories, hand search in specific targeted journals, harvest referencing and internet search engines.

The following databases will be searched to identity studies:

##### Electronic databases

- Applied Social Sciences Index and Abstracts (ProQuest)
- Criminal Justice Abstracts (EBSCO)
- Cumulative Index to Nursing and Allied Health Literature (CINAHL) (EBSCO)
- Dissertations and Theses Global (ProQuest)
- EMBASE (Ovid)
- National Criminal Justice Reference Service Abstracts (ProQuest)
- JSTOR
- Medline (Ovid)
- PsycInfo (Ovid)
- PubMed (Ovid)
- ClinicalTrials.gov
- International Clinical Trials Registry Platform (WHO): https://www.who.int/clinical-trials-registry-platform
- Conference Proceedings Index (Web of Science)
- Science Citation Index Expanded (Web of Science)
- Social Sciences Citation Index (Web of Science)
- Social Science Premium Collection (ProQuest)
- SCOPUS
- National Institute for Health and Care Research (NIHR): https://www.journalslibrary.nihr.ac.uk/#/
- ClinicalTrials.gov
- International Clinical Trials Registry Platform (WHO): https://www.who.int/clinical-trials-registry-platform

##### Governmental and grey literature search

Grey literature, such as policy documents and empirical reports from non‐academic sources, will be identified by searching the websites of relevant specialist organisations, including:

- United Nations https://www.un.org/
- UN Women https://www.unwomen.org/en
- World Health Organization https://www.who.int/
- The EU Fundamental Rights Agency https://fra.europa.eu/en
- Women Against Violence Europe https://wave-network.org/
- End Violence Women Against International https://evawintl.org/
- Global Network of Women's Shelters https://gnws.org/
- ANROWS https://www.anrows.org.au/
- Australian Institute of Criminology https://www.aic.gov.au/
- National Institute of Justice Crime Solutions https://crimesolutions.ojp.gov/
- What Works Crime Reduction Toolkit https://www.college.police.uk/research/crime-reduction-toolkit
- Her Majesty's Prison and Probation Service www.gov.uk
- Prison Research Centre https://www.prc.crim.cam.ac.uk/
- Correctional Service Canada https://www.csc-scc.gc.ca/index-en.shtml
- New Zealand Corrective Services https://www.corrections.govt.nz/
- The American Society of Criminology Division on Corrections and Sentencing https://ascdcs.org/
- Australian Institute of Criminology https://www.aic.gov.au/about-us
- Urban Institute https://www.urban.org/

Whilst noting that a limitation of this approach (mostly concerns of quality), we will include grey literature to decrease the likelihood of publication bias (Vevea & Woods, 2005) as well as contact relevant individuals and organisations for information about unpublished or ongoing studies. The use of non‐peer‐reviewed resources will be kept to a minimum and subject to quality appraisal.

##### Internet search

A web‐based search will be undertaken to identify further supplementary sources using Google Scholar. The Publish or Perish desktop app (https://harzing.com/resources/publish-or-perish) will be used to manage the results of Google Scholar searches in a systematic way for entry into EPPI‐ Reviewer.

##### Hand search

The table of contents of the relevant international journals will be searched to identify relevant primary studies eligible for inclusion. This includes:

- *Violence Against Women*;
- the *Journal of Interpersonal Violence*;
- the *International Journal on Criminology*;
- the *International Journal of Offender Therapy and Comparative Criminology*;
- *Journal of Experimental Criminology*;
- *Criminology*;
- *Journal of Aggression Maltreatment & Trauma*;
- *Journal of Interpersonal Violence*;
- *Journal of Quantitative Criminology*;
- *Trauma Violence & Abuse*.

We will undertake sufficient hand searching so that any recent publications that were not captured in the search are identified. We will therefore search the last two volumes before our search dates, of the journals highlighted.

##### Citation search

We will conduct citation searching on the studies identified for analysis, as well as searching the reference lists of systematic reviews identified in the database and internet searches detailed above. Citation searching in this regard means harvesting references from included studies.

### 3.7 Analysis and presentation

#### 3.7.1 Report structure

The EGM will include the following sections: an executive summary; background; objectives; methods; results; and discussion. We will include an executive summary to provide an overview of the EGM findings. We will explain the issue of current or historic DVA for mother in prison and on their release from prison in the background section, explaining how DVA and prison disrupt mothering and the mother–child relationship. This will provide context for why this EGM will describe DVA interventions in prison and for mothers on their release from prison. The methods section will describe the systematic search, including key search terms and search locations, along with the screening and data extraction processes. We will offer detail about the software programmes used to manage the evidence. We will provide an overview of the results including the number of eligible studies represented in a PRISMA diagram. The results will integrate the interactive EGM (see the earlier section on ‘Dimensions’). Finally, the discussion will reflect on the extent of evidence available and we will discuss gaps in the evidence highlighting future research recommendations and any implications for policy or practice. A plain English language statement of the EGM findings will be created.

#### 3.7.2 Filters for presentation

The EGM will present an interventions‐outcomes framework. A PRISMA flow diagram will be included and an online interactive matrix displaying the interactions between the interventions categories and outcomes. Presentation will use different colours to discern different types of studies (primary studies of effectiveness, qualitative studies). Searchable filters will include demographic information (age, ethnicity, asylum or migration status, disability), settings (prison, or community), geography (country or region), study design (RCT, non‐RCT, mixed method, qualitative) and intervention type (advocacy, psychosocial and skill‐building, therapeutic support, outreach, peer support, technology‐based intervention).

#### 3.7.3 Dependency

The unit of analysis for this EGM is the included studies. Where there are multiple papers published from the same study, the most recent open access publication will be included in this EGM. However, if previous publications of the same study include different outcome measures, these papers will be included only to report the missing outcomes. In this case, all papers will be treated as one single study. The EGM will list those studies with multiple papers clearly in the references. We will include data regarding the timing of follow‐up evaluations. Studies with multiple timepoint measurements following the intervention will be evident in the map.

### 3.8 Data collection and analysis

#### 3.8.1 Screening and study selection

As a first step, all titles and abstracts that are retrieved through the electronic searches will be downloaded to EPPI Reviewer. Duplicated references will be removed. To ascertain potential relevance for the review, following Polanin et al. (2019) guidelines, screening questions will include:

- Does the study report on an intervention/s that addresses DVA?
- Is the setting for the delivery of the intervention prison or post‐prison?
- Is the population of the study female prisoners in prison or post‐prison?

Post‐prison is defined as the 12 months following release from prison when individuals remain on license. To reduce potential bias, two reviewers will independently screen all titles and abstracts of references to identify primary studies to be classed as either ‘included’ or ‘excluded’ at this stage using the above criteria and recording decision‐making using an initial screening form. Discrepancies in screening decisions will be resolved through discussion, or, if necessary, by consulting the third member of the research team. We will document the selection of studies through the screening process in a PRISMA flow chart and record all decision‐making including the reasons for exclusion where relevant.

All titles/abstracts deemed potentially relevant during the initial screening will proceed to full‐text review. Two reviewers will independently review the full‐text versions using additional criteria to that employed in screening to reflect the other inclusion criteria regarding design, and outcomes. All results will be marked as ‘eligible’, ‘ineligible’, or ‘unsure’. All results marked as ‘unsure’ will be discussed among the reviewing team. Any discrepancies in review decisions will be resolved by discussion of the reviewing team.

#### 3.8.2 Data extraction and management

A data extraction and coding tool will be used to extract descriptive data from the studies to be included in the EGM (see Supporting Information: Appendix A Coding Sheet). Data to be extracted will include:

- General study details (document type, study location, year, funding).
- Participants (characteristics).
- Study details (research design, comparator—if relevant).
- Intervention (setting, agencies).
- Outcome type.

We will pilot the coding matrix with a random subset of publications to agree a coding scheme to guide the extraction and coding process for all included publications (Brown et al., 2003). Two reviewers will undertake data extraction with disagreements resolved by consensus. To check for inter‐rater reliability, 5% of each person's coding will be double‐coded by a second person. Where disagreements arise, a third review author will be consulted. Study authors will be contacted directly if documents are missing any pertinent information.

#### 3.8.3 Tools for assessing risk of bias/study quality of included reviews

Full‐text papers will be assessed for risk of bias and quality using the most appropriate tool (please note publications will not be rejected where the data quality is poor and unreliable albeit quality may be reported in the review on synthesis of findings). Quality appraisal and assessment of risk bias will be undertaken in an independent way by the same reviewers who completed data extraction, and discrepancies resolved as detailed above. We will use the most up‐to‐date versions of CASP tools for RCTS, cohort and qualitative studies (CASP, 2020) and the 2018 version of the Mixed Methods Assessment Tool (MMAT) to appraise all mixed‐methods studies (Hong et al., 2018). These tools were selected because all are designed to deal with quantitative, qualitative and mixed‐methods research within the same appraisal stage as part of a mixed‐methods systematic review. This will enable the appraisal of the methodological quality (including risk of bias) of five categories to studies: qualitative research; randomised controlled trials; non‐randomised studies; quantitative descriptive studies, and mixed methods studies. Where relevant, we will differentiate between different forms of bias (selection bias, attrition bias etc) reported in RCTs. As critical appraisal is about making judgments, two reviewers will independently appraise each study using the relevant CASP or MMAT tool. Discrepancies in judgements will be resolved through discussion, or, if necessary, by consulting the third member of the research team or colleague with methodological expertise relevant to the specific study. This will be reported in the review. We will use established criteria to appraise the quality of the study design using the PRISMA 2020 checklist (Page et al., 2021). Risk of bias ratings across eligible studies will be presented in tabular and narrative format.

#### 3.8.4 Methods for mapping

This EGM will use software developed by the EPPI Centre at the Social Science Research Centre, UCL. We will use EPPI‐Review 4 software to screen and code all studies for inclusion into the EGM (Thomas et al., 2010). The interactive map will be created using the EIPPI‐Mapper (Digital Solution Foundry of EPPI Centre, 2020).

## PLANS FOR UPDATING THE EGM

Three authors declare no conflicts of interest. Campbell is an editor for the Campbell Child and Young Persons Coordinating Group. She will have no influence on the editorial process for this protocol, or the EGM.

## SOURCES OF SUPPORT

**Internal sources**

• No sources of support provided

**External sources**

• No sources of support provided

## Supporting information

Supporting information.

## References

[cl21313-bib-0001] Apunyo, R. , White, H. , Otike, C. , Katairo, T. , Puerto, S. , Gardiner, D. , Kinengyere, A. A. , Eyers, J. , Saran, A. , & Obuku, E. A. (2022). Interventions to increase youth employment: An evidence and gap map [Interventions to increase youth employment: An evidence and gap map]. Campbell Systematic Review, 18(1), e1196. 10.1002/cl2.1196 PMC8847398 36913191

[cl21313-bib-0002] Baldwin, L. (2017). Tainted love. The impact of prison on maternal identity. Prison Service Journal, 233, 28–34.

[cl21313-bib-0003] Bronfenbrenner, U. (1979). The ecology of human development: Experiments by nature and design. Harvard University Press.

[cl21313-bib-0004] Brown, S. A. , Upchurch, S. L. , & Acton, G. J. (2003). A framework for developing a coding scheme for meta‐analysis. Western Journal of Nursing Research, 25(2), 205–222. 12666644 10.1177/0193945902250038

[cl21313-bib-0005] Casey‐Acevedo, K. , Bakken, T. , & Karle, A. (2004). Children visiting mothers in prison: The effects on mothers behaviour and disciplinary adjustment. Australian & New Zealand Journal of Criminology, 37(3), 418–430.

[cl21313-bib-0006] CASP . (2020). *Critical Skills Appraisal Programme*. CASP Checklist. CASP 2020.

[cl21313-bib-0007] Chartrand, C. , & Kilty, J. (2017). Corston principles in Canada: Creating the carceral Other and moving beyond women in prison. In L. Moore , P. Scraton , & A. Wahidin (Eds.), Women's imprisonment and the case for abolition. Critical reflections on Corston ten years on (pp. 109–128). Routledge.

[cl21313-bib-0008] Codd, H. (2020). International perspectives on mothering and imprisonment. In K. Lockwood (Ed.), Mothering from the Inside (pp. 181–196). Emerald Publishing.

[cl21313-bib-0009] Corston, J. (2007). *Review of women with particular vulnerabilities in the criminal justice system*. Home Office.

[cl21313-bib-0010] Crewe, B. , Hulley, S. , & Wright, S. (2017). The gendered pains of life imprisonment. British Journal of Criminology, 57, azw088.

[cl21313-bib-0011] Day, A. S. , & Gill, A. K. (2020). Applying intersectionality to partnerships between women's organizations and the criminal justice system in relation to domestic violence. The British Journal of Criminology, 60, 830–850.

[cl21313-bib-0012] Digital Solution Foundry of EPPI Centre . (2020). *EPPI Mapper Version 1.2.5* [Computer program]. Digital Solution Foundry of EPPI Centre.

[cl21313-bib-0013] Durfee, A. , & Goodmark, L. (2021). Is there a protection order to prison pipeline? Gendered dimensions of cross‐petitions. journal of aggression. Maltreatment & Trauma, 30(4), 471–490.

[cl21313-bib-0014] Enroos, R. (2011). Mothers in prison: Between the public institution and private family relations. Child & Family Social Work, 16, 12–21.

[cl21313-bib-0015] Ferrari, G. , Feder, G. , Agnew‐Davies, R. , Bailey, J. E. , Hollinghurst, S. , Howard, L. , Howarth, E. , Sardinha, L. , Sharp, D. , & Peters, T. J. (2018). Psychological advocacy towards healing (PATH): A randomized controlled trial of a psychological intervention in a domestic violence service setting. PLoS One, 13(11), e0205485. 30481183 10.1371/journal.pone.0205485 PMC6258512

[cl21313-bib-0016] Finer, L. (2020). Corston report. In F. Bernat , & K. Frailing (Eds.), The Encyclopedia of women and crime (pp. 121–123). Wiley & Sons.

[cl21313-bib-0017] Flynn, C. (2014). Getting there and being there: Visits to prisons in Victoria—The experiences of women prisoners and their children. Probation Journal, 61(2), 176–191.

[cl21313-bib-0018] Hong, Q. N. , Pluye, P. , Fàbregues, S. , Bartlett, G. , Boardman, F. , Cargo, M. , Dagenais, P. , Gagnon, M.‐P. , Griffiths, F. , Nicolau, B. , O'Cathain, A. , Rousseau, M.‐C. , & Vedel, I. (2018). *Mixed Methods Appraisal Tool (MMAT) (Version 2)*. Canadian Intellectual Property Office, Industry Canada.

[cl21313-bib-0019] Jones, M. S. (2020). Exploring coercive control, PTSD, and the use of physical violence in the pre‐prison heterosexual relationships of incarcerated women. Criminal Justice and Behavior, 47(10), 1299–1318.

[cl21313-bib-0020] Lockwood, K. (2017). Listening to mum: narratives of mothers in prison. In J. Woodiwiss , K. Smith , & K. Lockwood (Eds.), Feminist narrative research: Opportunities and challenges (1st ed., pp. 123–150). Palgrave Macmillan.

[cl21313-bib-0021] Macdonald, M. (2013). Women prisoners, mental health, violence and abuse. International Journal of Law and Psychiatry, 36(3–4), 293–303. 23642339 10.1016/j.ijlp.2013.04.014

[cl21313-bib-0022] McCauley, H. L. , Richie, F. , Hughes, S. , Johnson, J. E. , Zlotnick, C. , Rosen, R. K. , Wechsberg, W. M. , & Kuo, C. C. (2020). Trauma, power, and intimate relationships among women in prison. Violence Against Women, 26(6–7), 659–674. 30999810 10.1177/1077801219842948 PMC6800585

[cl21313-bib-0023] Mignon, S. , & Ransford, P. (2012). Mothers in prison: Maintaining connections with children. Soc Work Public Health, 27(1–2), 69–88. 22239379 10.1080/19371918.2012.630965

[cl21313-bib-0024] Ministry of Justice . (2012). *A distinct approach*.

[cl21313-bib-0025] Ministry of Justice . (2018). *Female offender strategy*.

[cl21313-bib-0026] Newman, C. , Fowler, C. , & Cashin, A. (2011). The development of a parenting program for incarcerated mothers in Australia: A review of prison‐based parenting programs. Contemporary Nurse, 39(1), 2–11. 21955261 10.5172/conu.2011.39.1.2

[cl21313-bib-0027] NOMS . (2015). Better outcomes for women offenders. National Offender Management Service.

[cl21313-bib-0028] Petrillo, M. (2021). ‘We've all got a big story’: Experiences of a trauma‐informed intervention in prison. The Howard Journal of Crime and Justice, 60(2), 232–250.

[cl21313-bib-0029] Page, M. J. , McKenzie, J. E. , Bossuyt, P. M. , Boutron, I. , Hoffmann, T. C. , Mulrow, C. D. , Shamseer, L. , Tetzlaff, J. M. , Akl, E. A. , Brennan, S. E. , Chou, R. , Glanville, J. , Grimshaw, J. M. , Hróbjartsson, A. , Lalu, M. M. , Li, T. , Loder, E. W. , Mayo‐Wilson, E. , McDonald, S. , … Moher, D. (2021). The PRISMA 2020 statement: An updated guideline for reporting systematic reviews. BMJ, 372, n71. 33782057 10.1136/bmj.n71 PMC8005924

[cl21313-bib-0030] Phelan, A. , & Kirwan, M. (2020). Contextualising missed care in two healthcare inquiries using a socio‐ecological systems approach. Journal of Clinical Nursing, 29(17–18), 3527–3540. 32564410 10.1111/jocn.15391

[cl21313-bib-0031] Polanin, J. R. , Pigott, T. D. , Espelage, D. L. , & Grotpeter, J. K. (2019). Best practice guidelines for abstract screening large‐evidence systematic reviews and meta‐analyses. Research Synthesis Methods, 10(3), 330–342.

[cl21313-bib-0032] Prison Reform International . (2020). *Addressing the 105,000 increase in the global female prison population, ten years after the Bangkok Rules were adopted*. https://www.penalreform.org/blog/addressing-the-105000-increase-in-the-global-female/

[cl21313-bib-0033] Prison Reform Trust . (2017). *“There's a reason we're in trouble”: Domestic abuse as a driver to women's offending*.

[cl21313-bib-0034] Roberts, J. (2019). ‘It was do or die’: how women's offending can occur as a by‐product of attempting to survive domestic abuse. Journal of Gender‐Based Violence, 3(3), 283–302.

[cl21313-bib-0035] Saran, A. , & White, H. (2018). Evidence and gap maps: A comparison of different approaches. Campbell Systematic Reviews, 14(1), 1–38. 10.4073/cmdp.2018.2 PMC8428058 37131398

[cl21313-bib-0036] Shuford, S. H. , Gjelsvik, A. , Clarke, J. , & van den Berg, J. J. (2018). Depression among women released from prison or jail in the United States. Journal of Health Care for the Poor and Underserved, 29(3), 914–929. 30122672 10.1353/hpu.2018.0068

[cl21313-bib-0037] Stark, E. (2007). Coercive control: The entrapment of women in personal life. Oxford University Press.

[cl21313-bib-0038] Stubbs, J. , & Baldry, E. (2017). In pursuit of fundamental change within the Australian penal landscape: Taking inspiration from the Corston Report. In L. Moore , P. Scraton , & A. Wahidin (Eds.), Women's imprisonment and the case for abolition (pp. 129–149). Routledge.

[cl21313-bib-0039] Sullivan, E. A. , Kendall, S. , Chang, S. , Baldry, E. , Zeki, R. , Gilles, M. , Wilson, M. , Butler, T. , Levy, M. , Wayland, S. , Cullen, P. , Jones, J. , & Sherwood, J. (2019). Aboriginal mothers in prison in Australia: A study of social, emotional and physical wellbeing. Australian and New Zealand Journal of Public Health, 43(3), 1753–6405. 10.1111/1753-6405.12892 30994971

[cl21313-bib-0040] Tallent, J. , Phillips, J. , & Coren, E. (2022). Protocol: Arts‐based interventions for offenders in secure criminal justice settings to improve rehabilitation outcomes: An evidence and gap map. Campbell Systematic Reviews, 18(3), e1255. 10.1002/cl2.1255 PMC9399450 36909891

[cl21313-bib-0041] Taylor, B. , & Sullivan, B. (2008). The Duluth model—What it is and is not: Clarifying and correcting common misconceptions. Journal for Women and Policing, 21(20), 33–37.

[cl21313-bib-0042] Thomas, J. , Brunton, J. , & Graziosi, S. (2010). *EPPI Reviewer 4: Software for research synthesis. Version 4* [Computer program]. Social Science Research Unit, EPPI Centre S, UCL, Institute of Education.

[cl21313-bib-0043] Trabold, N. , McMahon, J. , Alsobrooks, S. , Whitney, S. , & Mittal, M. (2020). A systematic review of intimate partner violence interventions: state of the field and implications for practitioners. Trauma, violence & abuse, 21(2), 311–325. 10.1177/1524838018767934 29649966

[cl21313-bib-0044] United Nations . (n.d.). What is domestic abuse . https://www.un.org/en/coronavirus/what-is-domestic-abuse

[cl21313-bib-0045] UNODC . (2014). Handbook on women and imprisonment (2nd ed.). United Nations.

[cl21313-bib-0046] Valenzuela‐Vela, L. , & Alcázar‐Campos, A. (2019). Gendered carceral logics in social work: The blurred boundaries in gender equality policies for imprisoned and battered women in Spain. Affilia, 35(1), 73–88.

[cl21313-bib-0047] Vevea, J. L. , & Woods, C. M. (2005). Publication bias in research synthesis. Psychological Methods, 10(4), 428–443. 16392998 10.1037/1082-989X.10.4.428

[cl21313-bib-0048] White, H. , Albers, B. , Gaarder, M. , Kornør, H. , Littell, J. , Marshall, Z. , Mathew, C. , Pigott, T. , Snilstveit, B. , Waddington, H. , & Welch, V. (2020). Guidance for producing a Campbell evidence and gap map. Campbell Systematic Reviews, 16(4), e1125. 10.1002/cl2.1125 PMC8356343 37016607

[cl21313-bib-0049] World Prison Brief . (2017). *World Female Imprisonment List*. https://www.prisonstudies.org/research-publications?shs_term_node_tid_depth=27

