# Supplementary material for: PROTOCOL: Domestic abuse interventions for mothers in or exiting prison: An evidence and gap map
Source: Campbell Syst Rev. 2023 Mar 2;19(1):e1313. doi: 10.1002/cl2.1313 (PMC9979188; doi:10.1002/cl2.1313)
Supplement: Supplementary file 1 — Supporting information. [file CL2-19-e1313-s001.docx]

# Appendices

## 1 Appendix A Coding Sheet

##

### General Study Details

| 1 | Study ID | Each document will be given a unique identifier |
| --- | --- | --- |
| 2 | Report ID | This will identify where there are multiple studies nested within the document (e.g., Rogers, 2022a, 2022b) |
| 3 | Type of document | Dropdown menu with the following options:   1. Peer-reviewed journal article 2. Book chapter 3. Dissertation/thesis 4. Conference presentation 5. Government report, technical report, or working paper 6. Other (give details) |
| 4 | Where was the intervention implemented (please list city, county, state/province and country as applicable)? |  |
| 5 | In what year was the intervention implemented? |  |
| 6 | Record the funding source if relevant |  |
| 7 | Publication status |  |
| 8 | Ongoing or completed? |  |

###

### Participants

| 1 | Who are the participants? | Dropdown menu with the following options:   1. Mothers in prison 2. Mothers recently exited prison 3. Both |
| --- | --- | --- |
| 2 | Characteristics of the sample | 1. Age (M, SD, range) 2. Ethnicity 3. Immigration status |
| 3 | Record any other relevant sample information. |  |

### Methodological Details

| 1 | Research design | 1. Randomised controlled trial 2. Quasi‐RCTs 3. Quasi‐experimental studies 4. Mixed method studies of all design types 5. Qualitative studies |
| --- | --- | --- |
| 2 | If relevant, what type of comparison condition was used? | 1. No treatment 2. Treatment-as-usual (specify in textbox) 3. Alternative treatment (specify in textbox) 4. Waitlist control 5. Other (specify in textbox) |

### Intervention Details

| 1 | What is the name of the intervention(s) as reported by study authors? |  |
| --- | --- | --- |
| 2 | What settings were used during the intervention(s)? | 1. Prison 2. Community-based (give detail) 3. Prison and community-based |
| 3 | Which agencies were involved in implementing the intervention? | Select all that apply   1. Police 2. Prison/correctional facilities 3. Probation/parole 4. Other criminal justice agency not listed here [specify] 5. Non-criminal justice partner* 6. If multiagency, who was involved? [textbox] |
| 4 | Does the intervention target DVA only, or is DVA one component of the intervention? | 1. DVA 2. One component |

### Outcome(s) Measurement*

*To be completed for each eligible outcome within a study (or group of reports for a study).

| 1 | Briefly describe the outcome being measured (e.g., its name, how it is conceptualised by study authors, what higher or lower value mean). |  |
| --- | --- | --- |
| 2 | What category does the outcome fall within? | Drop down menu:   1. DVA victimisation or revictimisation 2. Agency and self-efficacy in relation to relationships, conflict and abuse 3. Social and gender norms in relation to relationships, conflict and abuse 4. Risk and safety planning 5. Reoffending rates 6. Wellbeing (general or overall mental/emotional/psychological health) 7. Substance misuse 8. Safer accommodation 9. Mothering and parenting practice 10. Mother-child relationship 11. Acceptability of interventions 12. Other (specify in text box) |
| 3 | What category does the outcome fall within against socio-ecological framework | Drop down menu:     1. Individual 2. Community 3. Society |
| 4 | How is outcome measured | Drop down menu:     1. Official data (specify) 2. Self-report 3. Other (specify) |

This form has been informed by published coding forms (e.g., Sydes et al. 2022).
